# Associations between polymorphisms in folate-metabolizing genes and pancreatic cancer risk in Japanese subjects

**DOI:** 10.1186/s12876-016-0503-7

**Published:** 2016-07-29

**Authors:** Haruhisa Nakao, Kenji Wakai, Norimitsu Ishii, Yuji Kobayashi, Kiyoaki Ito, Masashi Yoneda, Mitsuru Mori, Masanori Nojima, Yasutoshi Kimura, Takao Endo, Masato Matsuyama, Hiroshi Ishii, Makoto Ueno, Sawako Kuruma, Naoto Egawa, Keitaro Matsuo, Satoyo Hosono, Shinichi Ohkawa, Kozue Nakamura, Akiko Tamakoshi, Mami Takahashi, Kazuaki Shimada, Takeshi Nishiyama, Shogo Kikuchi, Yingsong Lin

**Affiliations:** 1 Division of Gastroenterology, Department of Internal Medicine, Aichi Medical University School of Medicine, Nagakute, 480-1195 Japan; 2 Department of Preventive Medicine, Nagoya University Graduate School of Medicine, Nagoya, 466-8550 Japan; 3 Department of Public Health, Sapporo Medical University School of Medicine, Sapporo, 060-0061 Japan; 4 Department of Surgery, Surgical Oncology and Science, Sapporo Medical University, Sapporo, 060-8543 Japan; 5 Sapporo Shirakaba-dai Hospital, Sapporo, 062-0052 Japan; 6 Hepatobiliary and Pancreatic Section, Gastroenterological Division, Cancer Institute Hospital, Tokyo, 135-8550 Japan; 7 Clinical Research Center, National Hospital Organization Shikoku Cancer Center, Matsuyama, 791-0280 Japan; 8 Hepatobiliary and Pancreatic Medical Oncology Division, Kanagawa Cancer Center Hospital, Kanagawa, 241-8515 Japan; 9 Department of Internal Medicine, Tokyo Metropolitan Komagome Hospital, Tokyo, 113-8677 Japan; 10 Tokyo Metropolitan Otsuka Hospital, Tokyo, 170-8476 Japan; 11 Division of Molecular Medicine, Aichi Cancer Center Research Institute, Nagoya, 762-6111 Japan; 12 Department of Epidemiology, Nagoya University Graduate School of Medicine, Nagoya, 466-8550 Japan; 13 Division of Epidemiology and Prevention, Aichi Cancer Center Research Institute, Nagoya, 762-6111 Japan; 14 Department of Food and Nutrition, Gifu City Women’s College, Gifu, 501-2592 Japan; 15 Department of Public Health, Hokkaido University Graduate School of Medicine, Sapporo, 060-8638 Japan; 16 Central Animal Division, National Cancer Center Research Institute, Tokyo, 104-0045 Japan; 17 Department of Hepatobiliary and Pancreatic Surgery, National Cancer Center Hospital, Tokyo, 104-0045 Japan; 18 Department of Public Health, Aichi Medical University School of Medicine, Nagakute, 480-1195 Japan

**Keywords:** Folate, Polymorphism, Pancreatic cancer, Risk, Case-control study

## Abstract

**Background:**

Evidence supporting the associations between folate metabolizing gene polymorphisms and pancreatic cancer has been inconclusive. We examined their associations in a case-control study of Japanese subjects.

**Methods:**

Our case-control study involved 360 newly diagnosed pancreatic cancer cases and 400 frequency-matched, non-cancer control subjects. We genotyped four folate metabolizing gene polymorphisms, including two polymorphisms (rs1801133 and rs1801131) in the methylenetetrahydrofolate (*MTHFR*) gene, one polymorphism (rs1801394) in the 5-methyltetrahydrofolate-homocysteine methyltransferase reductase (*MTRR)* gene and one polymorphism (rs1805087) in the 5-methyltetrahydrofolate-homocysteine methyltransferase (*MTR)* gene. Genotyping was performed using Fluidigm SNPtype assays. Unconditional logistic regression methods were used to estimate odds ratios (ORs) and 95 % confidence intervals (CIs) for the associations between folate metabolizing gene variants and pancreatic cancer risk.

**Results:**

Overall we did not observe a significant association between these four genotypes and pancreatic cancer risk. For rs1801133, compared with individuals with the CC genotype of *MTHFR* C677T, the OR for those with the CT genotype and TT genotype was 0.87 (0.62-1.22) and 0.99 (0.65-1.51), respectively. For rs1801131, individuals with the CC genotype had approximately 1.2-fold increased risk compared with those with the AA genotype, but the association was not statistically significant. In analyses stratified by smoking and drinking status, no significant associations were noted for C677T genotypes. No significant interactions were observed with smoking and drinking with respect to pancreatic cancer risk.

**Conclusions:**

Our data did not support the hypothesis that *MTHFR* polymorphisms or other polymorphisms in the folate metabolizing pathway are associated with pancreatic cancer risk.

## Background

Pancreatic cancer is often diagnosed at an advanced stage and has the poorest 5-year survival rate of any cancer. There is no effective screening method to identify apparently healthy individuals who are at risk for pancreatic cancer. The etiology of pancreatic cancer remains largely unknown, with only cigarette smoking and long standing diabetes being established as risk factors [1]. The role of diet in carcinogenesis has been recognized for a long time. Evidence on the diet-pancreatic cancer associations from epidemiologic studies remains elusive, in part because of wide variation in dietary habits and the difficulty of accurate diet measurement. In nutrient-based studies, there has been much research interest in folate, a water soluble vitamin B that is abundant in green leafy vegetables, citrus fruit, legumes and cereals [2]. Folate plays a vital role in maintaining health because it is closely involved in two vital cellular processes, DNA methylation and DNA synthesis [2]. Epidemiologic studies have linked folate deficiency to a variety of conditions, including cardiovascular diseases and cancer [3–5]. For pancreatic cancer, previous studies have shown mixed findings on the association between dietary folate intake and pancreatic cancer risk [6–9], although a 2014 meta-analysis reported an inverse association [10]. One possible reason for the inconsistent findings is that folate status is determined by both dietary folate intake and folate metabolism, making it difficult to accurately quantify folate intake. Of numerous genes involved in the folate metabolizing pathway, *MTHFR* has been the most extensively studied [11, 12]. *MTHFR* irreversibly converts 5,10-methylenetetrahydrofolate to 5-methyltetrahydrofolate, the predominant form of folate in the circulation. Two variants in the *MTHFR*, namely C677T (rs1801133) and A1298C (rs1801131), have been the focus of most studies [11] because the variations are associated with a small change in protein structure. Compared with the CC genotype (wild-type), the TT genotype (minor homozygotes) of C677T has approximately 35 % lower enzyme activity and 10 % lower levels of methylhydrolate folate [11]. The 677 T variant has been associated with numerous conditions, including elevated homocysteine, spinal bifida, colon cancer, and Down syndrome [3] As for *MTHFR* A1298C, the homozygous CC genotype has approximately 60 % of normal *MTHFR* activity [12].

Evidence supporting the associations between folate metabolizing gene polymorphisms and pancreatic cancer has been inconclusive. To date, five case-control studies have examined the association between the polymorphisms in *MTHFR*, *MTR*, or *MTRR* genes and pancreatic cancer risk, with conflicting results [13–17]. Of them, two case-control studies conducted in Japan found no main effects for polymorphisms in *MTHFR* (rs1801133, rs1801131), *MTR* (rs1805087) or MTRR (rs1801394) genes [15, 17]. However, rs162049 in the *MTRR* gene, which encodes enzymes responsible for DNA methylation, has been shown to be associated with the susceptibility to pancreatic cancer in one previous Japanese case-control study [17]. The significance of these polymorphisms in the folate metabolizing pathway on pancreatic cancer remains to be determined.

Given that substantial evidence from epidemiologic and laboratory research supports an important role of folate in carcinogenesis [18], we genotyped several genetic polymorphisms in the folate metabolizing pathway and examined their associations with pancreatic cancer risk in Japanese subjects. We hypothesized that the variant *MTHFR* C667T and A1298C genotypes resulting in decreased enzyme activity are associated with an increased risk for pancreatic cancer. Additionally, we performed analyses stratified by cigarette smoking and alcohol drinking to address the possibility that these two lifestyle factors may modify the associations between genetic polymorphisms and pancreatic cancer risk.

## Methods

### Study subjects

This case-control study was designed to address the role of genetic variations in determining pancreatic cancer risk in Japanese subjects. A detailed description of the study method has been published elsewhere [19]. In brief, eligible cases were defined as newly diagnosed pancreatic cancer patients at five participating hospitals from April 1, 2010, through May 15, 2012. Those who had received chemotherapy for pancreatic cancer prior to the study entry were excluded. The diagnosis was based on imaging modalities and pathology reports (if available) were further reviewed for final diagnosis. Control subjects, who had no history of cancer, were recruited from inpatients and outpatients at each participating hospital, as well as from individuals who underwent medical checkups at one of the participating hospitals. The response rate was 85 % for cases and 98 % for control subjects. The control subjects were frequency matched to the case patients according to sex and age (within 10-year categories). Finally, 360 case patients and 400 control subjects were included in the present analysis. Approximately 90 % of tumors were histologically confirmed, with all tumors being adenocarcinomas.

For the main effect of genotypes, the sample size was estimated using the following assumptions: multiplicative genetic model, 10 % minor allele frequency, 0.1 % disease prevalence, 1:1 case-control ratio, and OR = 2.0. Then the number of cases required to achieve 90 % power at a significance threshold of α = 0.05 was 190 cases [20]. This study was conducted in accordance with the Helsinki Declaration. All the study subjects provided written informed consent. The Ethics Board of Aichi Medical University and the Institutional Review Board of all the participating hospitals approved this study.

### Data collection

A self-administered questionnaire was used to solicit detailed information on demographic characteristics, medical history, and lifestyle factors such as smoking, drinking and dietary habits. Dietary habits were surveyed using a validated food frequency questionnaire (FFQ), in which the study subjects were asked to describe the usual intake frequency of 36 foods during the previous year prior to the study entry [21]. After written informed consent was obtained from the study subjects, a 7 mL venous blood sample was collected. Genomic DNA was extracted using the same protocol for cases and controls, and subsequently stored at -30 °C until analysis.

### Genotyping assays

The genotyping of folate-metabolizing gene polymorphisms was performed using Fluidigm 192.24 Dynamic Array with BioMark HD Systems and EP1 (Fluidigm Corp., CA). SNPtype assay (Fluidigm Corp., CA), which employs allele-specifically designed fluorescences (FAM or VIC) primers and a common reverse primer, was used in this study. Genotype calls were obtained using the BioMark SNP Genotyping Analysis software. This software defined the genotype of each sample based on the relative intensities of fluorescences. The laboratory staff members were blinded to case or control status. Four quality control samples (negative control and positive controls for major homozygote, heterozygote and minor homozygote) were included in each assay, and the successful genotyping rate was 100 %.

### Statistical analysis

The differences in the characteristics of case patients and control subjects were tested using t-test, Mann-Whitney test, or chi-square test. The amount of daily ethanol intake was calculated based on the frequency and amount of alcohol beverages reported by the study subjects. The amount of daily dietary folate intake was estimated based on the responses to FFQ. A chi-square test was used to assess the Hardy-Weinberg equilibrium (HWE) in control subjects. Because the biological function of most SNPs has not been clearly defined, a co-dominant genomic model was assumed for SNP effects. Unconditional logistic regression models were used to estimate odds ratios (ORs) and 95 % confidence intervals (CIs) for the associations between folate metabolism-associated genetic polymorphisms and pancreatic cancer risk. All analyses were adjusted for age (continuous), sex (male or female), BMI (<20, 20-22.4, 22.5-24.9, or ≧25.0), and cigarette smoking (current, former, or never smokers). All tests were two-tailed; a P value less than 0.05 was used to define statistical significance. The interaction of genotype and smoking and drinking with regard to pancreatic cancer risk was assessed using a likelihood ratio test. All statistical analyses were performed using SAS 9.12 (SAS Institute, Inc., Cary, North Carolina, USA).

## Results

The distribution of genotypes for all SNPs among control subjects did not deviate from HWE. The selected characteristics of cases and controls are presented in Table 1. Compared with control subjects, the cases were more likely to have a history of diabetes and to be current smokers. The proportion of current drinkers is higher in control subjects than in case patients. The daily ethanol intake was 24.1 g/d among case patients and 17.5 g/d among control subjects. The median of daily folate intake was 338.9 μg for case patients and 359.5 μg for control subjects. High intake of dietary folate was inversely associated with pancreatic cancer risk, with OR of 0.52 (95 % CI: 0.33-0.82) among individuals falling into the highest quartile when compare with those falling into the lowest quartile.

**Table 1:** Characteristics of case patients and control subjects

Characteristics	Case patients	Control subjects	P
	(*N* = 360)	(*N* = 400)	
Age (mean ± SD)	67.8 ± 8.8	64.8 ± 9.5	<0.0001
Male, *N* (%)	215 (59.7)	226 (56.5)	0.58
Body mass index (kg/m2) (mean ± SD)	22.9 ± 3.3	22.8 ± 3.2	0.62
History of diabetes, *N* (%)			<0.0001
No	269 (74.7)	362 (90.5)	
Yes	87 (24.2)	35 (8.7)	
Unknown	4 (1.1)	3 (0.8)	
Smoking status, *N* (%)			<0.0001
Non-smokers	145 (40.2)	202 (50.5)	
Former smokers	119 (33.1)	140 (35.0)	
Current Smokers	96 (26.7)	58 (14.5)	
Alcohol drinking, *N* (%)			0.23
Non-drinkers	134 (37.2)	147 (36.8)	
Former drinkers	24(6.7)	16 (4.0)	
Current Drinkers	202(56.1)	237(59.2)	
Ethanol intake (g/d), median (25th, 75th percentile)	24.1 (7.8, 48.9)	17.5 (5.7, 43.4)	0.02
Dietary folate intake (μg), median (25th, 75th percentile)	338.9 (280.9, 407.6)	359.2 (293.0, 438.8)	0.0007

SD, standard deviation

Table 2 shows the ORs of pancreatic cancer in relation to individual polymorphisms in the following genes: *MTHFR (*rs1801133, rs1801131), *MTRR* (rs1801394), and *MTR* (rs1805087). Overall no significant associations were noted between any single genotype and risk of pancreatic cancer. For rs1801133, compared with individuals with the CC genotype of *MTHFR* C677T, the OR for those with the CT genotype and TT genotype was 0.87 (0.62–1.22) and 0.99 (0.65–1.51), respectively. For rs1801131, individuals with the CC genotype had approximately 1.2-fold increased risk compared with those with the AA genotype, but the association was not statistically significant.

**Table 2:** Associations of pancreatic cancer with folate metabolizing gene polymorphisms

	Case patients (*n* = 360)	Control subjects (*n* = 400)	Crude OR	95 % CI	Multivariable-adjusted OR*	95 % CI	P for trend**
*MTHFR*_C677T rs1801133							
CC	127	124	1.00		1.00		
CT	161	194	0.81	0.59-1.12	0.87	0.62-1.22	
TT	72	82	0.86	0.57-1.28	0.99	0.65-1.51	0.86
*MTHFR*_A1298C rs1801131							
AA	240	285	1.00		1.00		
AC	107	102	1.25	0.90-1.72	1.28	0.91-1.80	
CC	13	13	1.19	0.54-2.61	1.22	0.53-2.80	0.17
*MTRR*_A66G rs1801394							
AA	167	206	1.00		1.00		
AG	157	158	1.23	0.91-1.66	1.29	0.94-1.77	
GG	36	36	1.23	0.74-2.04	1.12	0.66-1.91	0.27
*MTR*_A2756G rs1805087							
AA	224	241	1.00		1.00		
AG	117	142	0.89	0.65-1.20	0.87	0.63-1.21	
GG	19	17	1.20	0.61-2.37	1.53	0.75-3.12	0.89

*OR* odds ratio, *CI* confidence interval

*OR was adjusted for age, sex, cigarette smoking, BMI, and history of diabetes

**P for trend is shown for multivariable-adjusted OR

Table 3 shows the associations of pancreatic cancer with folate metabolizing gene polymorphisms by smoking and drinking status. There were no significant associations in either never smokers or current smokers. Similarly, there were no significant associations in either never drinkers or current drinkers.

**Table 3:** Associations of pancreatic cancer with polymorphisms in genes involved in folate metabolism by smoking and drinking status

	Case patients (*n* = 360)	Control subjects (*n* = 400)	Multivariable-adjusted OR	95 % CI	Casepatients (*n* = 360)	Control subjects (*n* = 400)	Multivariable-adjusted OR	95 % CI	P for interaction
*MTHFR*_C677T rs1801133	Never smokers			Ever smokers					
CC	48	63	1.00		79	61	1.00		
CT	61	89	0.83	0.49-1.40	100	105	0.80	0.51-1.26	
TT	36	50	0.91	0.50-1.66	36	32	1.02	0.55-1.87	0.94
*MTHFR*_A1298C rs1801131									
AA	95	143	1.00		145	142	1.00		
AC	45	53	1.39	0.84-2.29	62	49	1.24	0.78-1.96	
CC	5	6	1.71	0.49-6.00	8	7	1.12	0.38-3.28	0.83
*MTRR*_A66G rs1801394									
AA	61	101	1.00		106	105	1.00		
AG	69	82	1.37	0.86-2.18	88	76	1.16	0.75-1.79	
GG	15	19	1.21	0.56-2.62	21	17	1.09	0.52-2.26	0.46
*MTR*_A2756G rs1805087									
AA	98	127	1.00		126	114	1.00		
AG	41	69	0.73	0.44-1.19	76	73	1.08	0.70-1.66	
GG	6	6	1.61	0.48-5.40	13	11	1.35	0.56-3.24	0.93
*MTHFR*_C677T rs1801133	Never drinkers			Ever drinkers					
CC	47	54	1.00		80	70	1.00		
CT	58	66	1.05	0.60-1.82	103	128	0.74	0.48-1.16	
TT	29	27	1.33	0.67-2.64	43	55	0.81	0.47-1.40	0.41
*MTHFR*_A1298C rs1801131									
AA	88	101	1.00		152	184	1.00		
AC	39	40	1.16	0.67-2.01	68	62	1.40	0.90-2.16	
CC	7	6	1.43	0.44-4.61	6	7	1.06	0.32-3.55	0.78
*MTRR*_A66G rs1801394									
AA	58	67	1.00		109	139	1.00		
AG	56	66	1.03	0.61-1.73	101	92	1.45	0.96-2.19	
GG	20	14	1.61	0.73-3.56	16	22	0.78	0.37-1.65	0.15
*MTR*_A2756G rs1805087									
AA	87	86	1.00		137	155	1.00		
AG	43	57	0.78	0.47-1.32	74	85	0.98	0.64-1.50	
GG	4	4	1.49	0.35-6.38	15	13	1.59	0.69-3.65	0.57

OR was adjusted for age, sex, BMI and history of diabetes

Table 4 shows the joint effects of smoking, drinking and *MTHFR* genotypes on pancreatic cancer risk. No significant interactions were observed for C667T genotypes and cigarette smoking; the OR of pancreatic cancer was 1.94 (1.05–3.57) for individuals who had the TT genotype and were ever smokers. Similarly, no significant interactions were noted for C677T genotypes and alcohol drinking; the OR of pancreatic cancer was 1.05 (95 % CI: 0.62-1.78) for individuals who had the TT genotype and were ever smokers.

**Table 4:** Joint effects of smoking, drinking and MTHFR C677T on pancreatic cancer risk

C677T Genotype	Smoking	Case patients	Control subjects	OR (95 % CI)
CC + CT	Never	109	152	1.00
TT	Never	36	50	1.04 (0.62-1.73)
CC + CT	Ever	179	166	1.69 (1.13-2.54)
TT	Ever	36	32	1.94 (1.05-3.57)
				P for interaction = 0.79
	Drinking	Case patients	Control subjects	OR* (95 % CI)
CC + CT	Never	105	120	1.00
TT	Never	29	27	1.33 (0.72-2.45)
CC + CT	Ever	183	198	1.10 (0.75-1.61)
TT	Ever	43	55	1.05 (0.62-1.78)
				P for interaction = 0.38

*OR* odds ratio, *CI* confidence interval

*OR was adjusted for age, sex, body mass index, cigarette smoking and history of diabetes

## Discussion

In this genetic case-control association study, we found no statistically significant associations between polymorphisms in folate metabolizing gene pathways, including *MTHFR* C677T and A1298C, and the risk of pancreatic cancer. Previous studies on the associations of *MTHFR* (C677T) with pancreatic cancer risk have shown inconsistent results [13–16]. A hospital-based case-control study, conducted at the MD Anderson Cancer Center in the United States, found that individuals with the TT variant genotype had a 2-fold increased risk for pancreatic cancer when compared with individuals with the CC genotype [13]. A stronger association was reported in a case-control study of Chinese population, in which the OR was 5.12 for individuals with the TT genotype compared with individuals with the CC genotype [14]. By contrast, two previous case-control studies in Japan did not find significant main effects for *MTHFR* C677T and A1298C genotypes [15, 17]. Our null findings were in agreement with results of these two previous studies. Our null findings were also consistent with the conclusion of a recent meta-analysis, which included all previous studies and showed no significant main effects for both *MTHFR* C677T and A1298C genotypes [22]. Furthermore, a recent study has sought to examine 37 genes and 834 SNPs related to one-carbon metabolism. There were no significant associations for any of the SNPs after correction for multiple comparisons [23]. However, in the case-control study by Ohnami et al, rs162049 (intronic SNP) in the *MTRR* gene showed significant associations after multiple testing [17]. One strength of their study is that functional tests were performed to collaborate the SNP-phenotype association. Unfortunately, we did not genotype rs162049 in the *MTRR* gene, but further studies needs to replicate their positive finding.

Given the lower enzymatic activity in individuals with the variant TT genotype of *MTHFR* C677T, a key enzyme in the folate metabolizing pathway, we hypothesized that the TT genotype carriers have an increased risk of pancreatic cancer. Our results, however, showed that case patients had a similar distribution of the TT genotypes with that of control subjects, and neither CT nor TT genotypes were significantly associated with the risk when compared with CC genotypes. The reason for our null findings on the associations between *MTHFR* genotypes and pancreatic cancer risk is not clear, but the differences observed in minor allele frequencies among ethnic groups may in part account for the conflicting results. The genotype frequencies of *MTHFR* C677T differed between our case-control study and the previous case-control study that involved a US population [13]. Even though there may be no main effects for the genotypes themselves, previous studies have shown that the *MTHFR* 677C → T polymorphism is only associated with increased coronary heart disease risk in a setting of low folate intake [4]. This finding suggested that the effect modification by dietary folate intake is important when interpreting the genotype-disease associations. If nutritional folate status is poor, the 677 T variant might promote the misincorporation of uracil into DNA, leading to genomic instability, a hallmark of cancer [3]. This mechanism may, in part, account for the increased cancer risk among individuals with the 677 T variant of *MTHFR*. On the other hand, if folate intake is adequate, the 677 T variant of *MTHFR* preferentially routes one-carbon units to DNA synthesis at the expense of methionine, which is involved in DNA methylation [3]. To address the possible effect modification by dietary folate intake, we evaluated their associations in low- versus high- dietary intake group, and found no significant effect modifications (data not shown). One possible reason is that most subjects in our study had adequate dietary folate intake because the estimated amount was comparable to 240 μg per day for Japanese adults recommended by the government.

In addition to dietary folate intake, other lifestyle factors that may modify the associations between *MTHFR* C677T and pancreatic cancer risk are smoking and drinking. Because alcohol is known as a folate antagonist and because smoking may impair folate status, several studies have evaluated the joint effects of the *MTHFR* polymorphisms with cigarette smoking or alcohol consumption in relation to pancreatic cancer risk [13–15]. In one hospital-based case-control study conducted in Japan, the risk of pancreatic cancer increased by 4.5-fold among heavy drinkers with the *MTHFR* 677 CC genotype [15]. Similarly, another two case-control studies found that individuals with the 677 T variant combined with heavy smoking or drinking had significantly increased risk for pancreatic cancer [13, 14]. In contrast to their findings, we failed to observe the synergistic effect of the genotypes with either cigarette smoking or heavy alcohol consumption. It is worth noting that the analysis of gene-environment interaction was limited by a small sample size in our study, as well as in those previous studies. So the probability that the positive interaction was due to chance cannot be ruled out.

Our study has several limitations. First, we had sufficient number of cases required to detect significant associations for the main effect of genotypes based on sample size estimation; however, we did not have sufficient power to detect significant associations in the analyses examining the synergistic effect of genotypes and exposure of interest. Assuming a dominant genetic model, a dichotomous exposure prevalence of 10 %, a relative risk for a genotype of 1.5, a relative risk for exposure of 1.5, and 1:1 case-control ratio, we need thousands of cases and controls to detect multiplicative interactions with relative risk of 2.0 [24]. Second, environmental exposure (folate) assessment or environmental exposure (folate)-pancreatic cancer association could have been affected by selection or recall bias inherent in case-control studies; however, the genotype-pancreatic cancer association was generally not affected by bias due to differential environmental exposure assessment between cases and control subjects. Third, the lack of the data on plasma folate levels did not allow us to address the relationship among *MTHFR* genotypes, plasma levels and pancreatic cancer risk. It has been shown that DNA methylation was affected by genotype among only those with lower plasma folate levels [25]. Moreover, it is expected that multifactorial interactions may exist among *MTHFR* genotypes, dietary folate, circulating folate level, alcohol, and/or other relevant nutrients including vitamin B2 and B12. Further studies need to develop a novel analytical method addressing the complex gene-environment interaction pathways involved in folate-derived carcinogenesis.

## Conclusions

In conclusion, our case-control study suggests that there is no association between genetic polymorphisms in folate-metabolizing genes and the risk of pancreatic cancer in Japanese subjects. The results may not be generalizable in view of limited sample size. Our study is also limited to address effect modification by alcohol, smoking or folate intake (gene-environment interaction) because of inadequate statistical power.

## Abbreviations

BMI, body mass index; CI, confidence interval; FFQ, food frequency questionnaire; HWE, Hardy-Weinberg equilibrium; *MTHFR*, methylenetetrahydrofolate; MTR, 5-methyltetrahydrofolate-homocysteine methyltransferase; *MTRR*, 5-methyltetrahydrofolate-homocysteine methyltransferase reductase; ORs, odds ratios.
